# Cardiac Tissue Engineering: A Journey from Scaffold Fabrication to In Vitro Characterization

**DOI:** 10.1002/smsc.202400079

**Published:** 2024-07-22

**Authors:** Farinaz Ketabat, Jane Alcorn, Michael E. Kelly, Ildiko Badea, Xiongbiao Chen

**Affiliations:** ^1^ Division of Biomedical Engineering University of Saskatchewan 57 Campus Drive Saskatoon S7N 5A9 Canada; ^2^ College of Pharmacy and Nutrition University of Saskatchewan 107 Wiggins Road Saskatoon S7N 5E5 Saskatchewan Canada; ^3^ Department of Surgery, College of Medicine University of Saskatchewan 107 Wiggins Road Saskatoon S7N 5E5 Canada; ^4^ Department of Mechanical Engineering University of Saskatchewan 57 Campus Drive Saskatoon S7N 5A9 Canada

**Keywords:** 3D‐printing, biomaterials, cell types, electrospinning, manufacturing techniques

## Abstract

Cardiac tissue engineering has been rapidly evolving with diverse applications, ranging from the repair of fibrotic tissue caused by “adverse remodeling,” to the replacement of specific segments of heart tissue, and ultimately to the creation of a whole heart. The repair or replacement of cardiac tissue often involves the development of tissue scaffolds or constructs and the subsequent assessment of their performance and functionality. For this, the design and/or selection of biomaterials, and cell types, scaffold fabrication, and in vitro characterizations are the first starting points, yet critical, to ensure success in subsequent implantation in vivo. This highlights the importance of scaffold fabrication and in vitro experiments/characterization with protocols for cardiac tissue engineering. Yet, a comprehensive and critical review of these has not been established and documented. As inspired, herein, the latest development and advances in scaffold fabrication and in vitro characterization for cardiac tissue engineering are critically reviewed, with focus on biomaterials, cell types, additive manufacturing techniques for scaffold fabrication, and common in vitro characterization techniques or methods. This article would be of benefit to the ones who are working on cardiac tissue engineering by providing insights into the scaffold fabrication and in vitro investigations.

## Graphical abstract

This article comprehensively addresses advancements in the development of engineered cardiac tissues using additive manufacturing techniques. The key aspects covered include: 1) necessary componentsfor developing cardiac tissue, providing a foundation for subsequent discussions; 2) cutting‐edge additive manufacturing techniques; and 3) characterization techniques crucial for ensuring the functionality of cardiac tissue before embarking on in vivo studies.

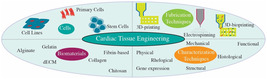

## 1 Introduction

Cardiac tissue engineering (CTE) is a promising approach that may overcome the shortcomings of traditional treatments for heart disease. CTE aims to engineer scaffolds or constructs mimicking the myocardium to repair cardiac tissue damage and ultimately replace the fibrotic scar tissue with functional and contractile cardiac tissue.^[^ ^1^, ^2^, ^3^ ^]^ For a successful CTE application, scaffold fabrication is critically important and typically involves three components, i.e., 1) biomaterials, 2) specific cells to create cardiac tissue architecture, and 3) techniques to fabricate the tissue scaffolds from biomaterials and cells. Once fabricated, the scaffolds are either implanted/transplanted in vivo immediately, or grown in vitro for more maturation and then transplanted. To enhance the likelihood of a successful transplantation, it is imperative to perform a comprehensive in vitro examination and analysis on the engineered cardiac tissue scaffold prior to its implantation. However, the literature currently lacks the established and essential protocols for such in vitro experiments and examination before progressing to the in vivo phase. In this article, we briefly, yet critically, review the latest developments and advances in biomaterials, cell types, and fabrication techniques used for CTE and furthermore discuss various in vitro characterization methods that are essential for evaluating the engineered cardiac constructs.

## 2 Biomaterials and Cells for CTE

CTE approaches are generally based on three main components, i.e., the biomaterials and architecture of scaffolds, the cells that form cardiac tissue, and fabrication techniques. A variety of biomaterials have been utilized to engineer scaffolds that simulate the complex and dynamic microenvironment of the native heart.^[^ ^4^ ^]^ These engineered scaffolds provide a supportive environment for cell growth into cardiac tissue. The human heart is composed of ≈2–3 billion cardiac muscle cells while a population of noncardiac muscle cells exceeds cardiac cells by more than threefold. The noncardiac cells include smooth muscle cells (SMCs), endothelial cells (ECs), fibroblasts, connective tissue cells, immune cells, and a limited number of pluripotent cardiac stem cells.^[^ ^5^ ^]^ Therefore, a successful cardiac tissue construct must employ a diverse array of cells that mimic the natural distribution found in the human heart. Hence, the development of advanced fabrication techniques for creating scaffolds or cardiac constructs becomes essential.

### 2.1 Biomaterials for Fabrication of Scaffolds

Scaffolds for CTE are commonly 3D structures composed of biomaterials that can imitate the extracellular matrix (ECM) in the heart. Recently, studies have employed combinations of biomaterials to create such scaffolds. Alginate, a biocompatible natural hydrogel, shares characteristics with the ECM.^[^ ^6^ ^]^ As a nonimmunogenic polymer, alginate has gained considerable attention in tissue engineering, particularly in CTE, owing to its favorable properties and cost‐effectiveness.^[^ ^6^, ^7^ ^]^ However, using alginate alone for CTE is not recommended due to its limited ability to encourage fibronectin adhesion;^[^ ^6^ ^]^ fibronectin is an essential protein involved in cell behavior, such as adhesion and migration.^[^ ^8^ ^]^ To overcome this limitation, alginate is combined with various materials, such as gelatin,^[^ ^6^, ^9^, ^10^, ^11^ ^]^ fibrinogen,^[^ ^12^, ^13^, ^14^ ^]^ chitosan,^[^ ^15^ ^]^ or collagen,^[^ ^16^ ^]^ for CTE applications. Although these biomaterials may afford some advantages, each has their own sets of limitations (**Table** **1**). A developing area of investigation is the use of heart tissue‐derived decellularized ECM (dECM) hydrogels. dECM hydrogels can mimic the complex biological and biochemical microenvironment of the cardiac tissue through the recreation of the ECM composition of cardiac tissue, which may overcome the limitations posed by other biomaterials.^[^ ^17^ ^]^ However, preparation of dECM remains challenging with a need to overcome issues such as the presence of residual DNA, which could trigger an inflammatory reaction upon transplantation, as well as the preservation of ECM proteins during the cell extraction process throughout the production of dECM.^[^ ^18^ ^]^

**Table 1:** Advantages and disadvantages of biomaterials for CTE.

Biomaterial	Advantages	Disadvantages	References
Alginate	High biocompatibility, biodegradability, nonthrombogenicity, hydrophilicity, low cost	Poor cell adhesion and proliferation leading to weakened cell regeneration	[92, 93]
Gelatin	High biocompatibility, nonimmunogenicity, high biodegradability, containing natural receptors for cell adhesion	Temperature sensitive	[92]
Fibrinogen/fibrin‐based biomaterials	Promoting cell proliferation, cell migration and cell differentiation, speeding up wound healing, reducing the infarct size, increasing the survival rate of transplanted cells, increasing the blood supply to the damaged tissue	Gel shrinkage, poor mechanical properties, fibrin deformation, the risk of disease transmission	[93]
Chitosan	High blood compatibility, cytocompatibility, biocompatibility, low cost, biodegradability, antibacterial and antifungal properties	Not suitable for bioprinting living cells, slow gelation rate, poor mechanical properties	[92, 93]
Collagen	The principal component of ECM, having natural receptors for cell adhesion, and differentiation, low antigenicity, high biocompatibility and bioactivity, modifiable biodegradability	Poor mechanical properties and low elasticity	[92, 93]
dECM	Containing a variety of biomolecules and growth factors, highly supporting cell growth and cell functions	Poor mechanical properties, risk of containing toxic residual following decellularization	[94]

### 2.2 Cells

Four types of cells form the structure of the heart: cardiomyocytes (CMs), ECs, SMCs, and cardiac fibroblasts (CFs). Heart tissue also contains other types of cells, such as immune cells, derived from other parts of the body and migrates to the heart to fulfill their roles in organ surveillance and tissue maintenance.^[^ ^19^ ^]^ For the purpose of cardiac tissue constructs, CTE research encompasses cells classified into three groups: immortalized cell lines, primary cells, and stem cells (**Table** **2**). Each category has its own advantages and shortcomings, highlighting their unique benefits and limitations within the context of CTE applications, as summarized in Table 2.

**Table 2:** Different cell types used in CTE (ESCs: embryonic stem cells; MSCs: mesenchymal stem cells).

Cells		Origin	Advantages	Disadvantages	Relevant References
Immortalized cell lines	H9c2	Undifferentiated myoblasts, from the rat left ventricle with embryonic origin	A better model to simulate ischemic reperfusion injury, show higher mitochondria content, higher citrate synthase activity, elevated oxidative activity, greater ATP synthesis, enhanced efficiency of energy transfer, more organized regulation compared to other cardiac cell lines	No contractility	[95]
	HL‐1	Adult mouse atrium	May contract	May lose the contractility after few passages	[95]
	C2C12	Mouse muscle	Capable of undergoing a sequential differentiation process, autologous muscle biopsies can be used without the need for using immunosuppression, the absence of risk of malignant transformation, higher resistance to ischemia in comparison with cardiac muscle.	Not authentic CMs	[96, 97]
Primary cells	hPCMs	Adult human primary CMs	Possess all native physiological and pharmacological features of mature CMs	Susceptible to biochemical and mechanical perturbations, challenging culture and cryopreservation, limited availability of donor heart tissue, variability of donor tissue	[98, 99]
	NVRM	Neonatal ventricular rat CMs	Forming spontaneously beating cells 20 hrs after plating, long‐term longevity, maturation, gap‐junction establishment with host CMs, enhanced heart function upon transplantation	Different morphology compared to adult CMs, ethical concernsregarding large‐scale harvesting	[100, 101, 102]
	HUVECs	Human umbilical vein ECs from biological waste after child's birth	Capable of being cocultured with other cells such as neonatal rat CMs, easy to isolate	Might not fully represent the diversity of ECs for vasculogenesis, not suitable for long‐term studies	[26, 103, 104, 105]
Stem cells	ESCs	Derived from embryos	Pluripotent, expand extensively in vitro	Ethical concerns regarding their isolation	[106]
	MSCs	Derived from the ectoderm and mesoderm and found in multiple sources such as adult bone marrow, adipose tissue, etc.	Multipotent, highly proliferative, easy collection, short culture period	Might get tumorigenic, poor survival rate, low retention rate	[106, 107, 108]
	iPSC	Derived from adult somatic cells and reprogrammed to ESC‐like state	Pluripotent, human origin, easily accessible, proliferative, less ethical concerns compared to ECSs	Risk of tumorigenicity	[106, 109, 110, 111]

## 3 Fabrication Techniques for Cardiac Tissue Scaffolds

Tissue engineering applications have employed various technologies to fabricate scaffolds, but mainly have focused on developing porous 3D structures. Most successful scaffold fabrication techniques are those with ability to produce scaffolds in large quantities, high quality, and consistency.^[^ ^20^ ^]^ Many conventional and more advanced fabrication techniques exist for developing cardiac scaffolds. This review concentrates on additive manufacturing (AM)/3D‐printing and electrospinning techniques, owing to their exceptional versatility.

Generally, tissue scaffolds can be engineered through two major approaches: 1) Traditional (top‐down): in this approach, porous scaffolds are first constructed, and then the target cells and/or other required biomolecules are seeded onto the scaffolds.^[^ ^21^ ^]^ 2) Modular (bottom‐up): this method involves use of cell‐laden materials, with or without the target biomolecules such as growth factors, to assemble the tissue constructs.^[^ ^21^ ^]^

In the top‐down technique, target cells are seeded onto the 3D scaffolds to attach, proliferate, and fully incorporate into the biodegradable structure to eventually form ECM. However, this method faces two major challenges: first, the scaffolds often replicate the external shape and size of the tissue rather than its internal complex microenvironment; second, due to lack of vascularization, constructing complex and functional tissues becomes challenging.^[^ ^21^, ^22^ ^]^ To address these challenges, the bottom‐up technique has emerged in which individual building blocks are assembled from bottom up to fabricate complex structures. In this method, the shape and composition of each individual building block can be controlled. Additionally, a greater cell density and vascularization could be achieved, which are vital for a successful engineered tissue, especially cardiac tissue.^[^ ^22^ ^]^ However, tissue constructs are still fabricated using both top‐down and bottom‐up techniques or a combination of both.

**Figure 1:**
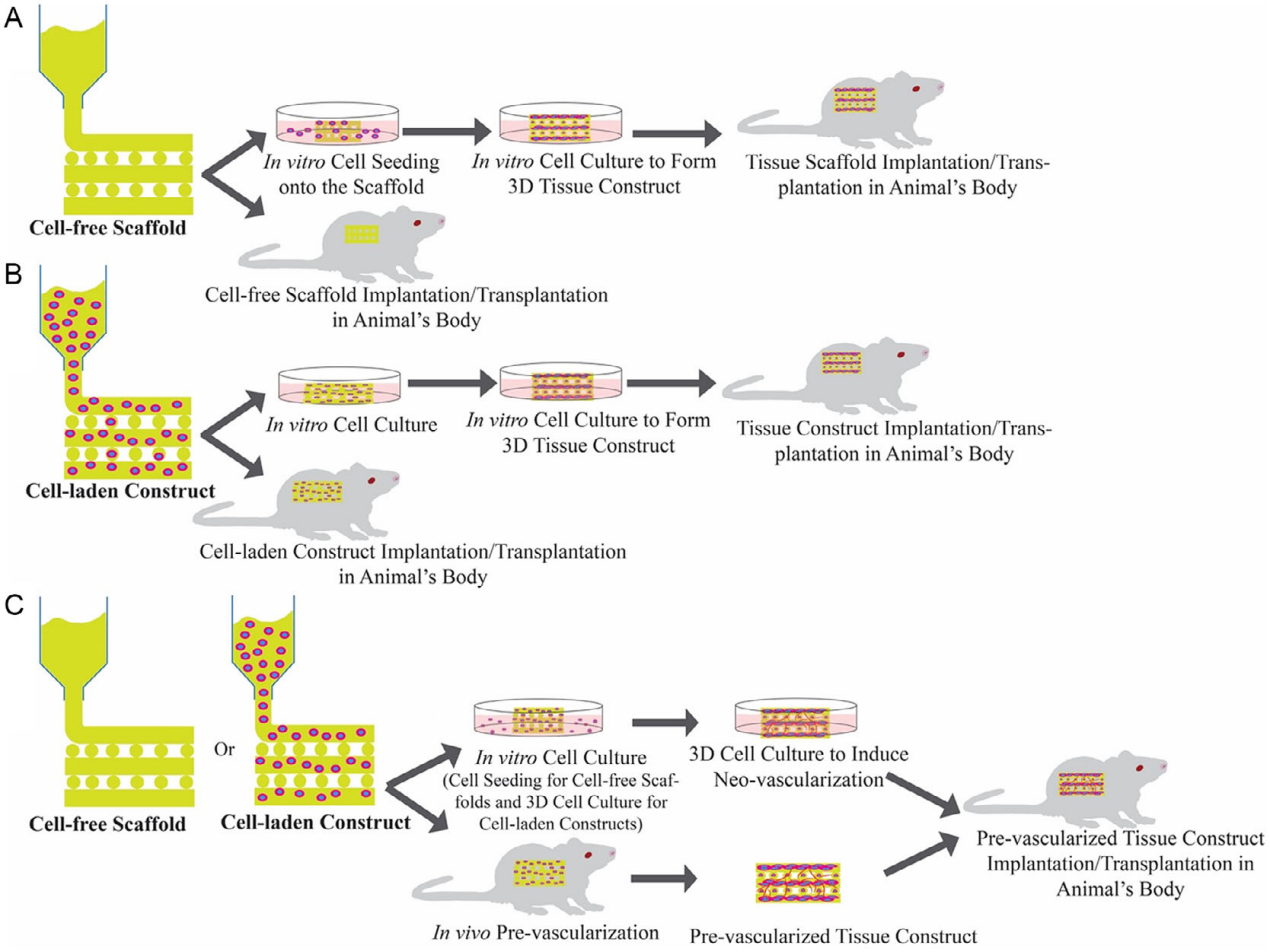
Different approaches for 3D‐printing tissue scaffolds: A) cell‐free scaffolds: materials without cells are 3D‐printed, and the 3D‐printed scaffolds are either used for in vivo studies or first cultured in vitro to grow cells onto/into the scaffold before implantation; B) cell‐laden constructs: materials with cells are 3D‐printed, and then they are either used directly for in vivo studies or they are cultured first to form a 3D tissue construct prior to in vivo studies; and C) pre‐vascularized constructs: cell‐free or cell‐laden scaffolds are 3D‐printed, cultured in vitro to form neovascularization, and then are transplanted into the animal.

AM technologies have been exploited in the development of more complex tissues and organs as a substitute for conventional methods. Among AM technologies, 3D‐printing is a promising approach which deposits materials in a layer‐by‐layer fashion in 3D space using a designed computer model file. This technique offers opportunity to combine materials with living cells to print more functional tissue constructs (3D‐bioprinting).^[^ ^23^ ^]^ Besides 3D‐printing, electrospinning is a progressing and promising tissue engineering application. This technique uses high‐voltage electric field to deposit nanofibers and has been employed in many studies focused on vascular and CTE.^[^ ^24^ ^]^ To our knowledge, AM technologies, especially 3D‐printing techniques, have been used to manufacture three types of bioengineered scaffolds, including those designed for cardiac tissue applications: 1) cell‐free scaffolds, 2) cell‐laden constructs, and 3) pre‐vascularized constructs (**Figure** **1**).

### 3.1 Cell‐Free Scaffolds

Cell‐free scaffolds have important advantages for CTE. Such scaffolds overcome the time and cost required to harvest, proliferate, and differentiate the cells associated with other tissue fabrication techniques. Furthermore, cell‐free scaffolds are more likely to be regarded as medical devices by regulatory agencies such as the Food and Drug Administration (FDA), thus reducing the time and costs associated with regulatory approval.^[^ ^25^ ^]^ Although the cell‐free scaffold eliminates the complexity of 3D‐bioprinting, it might lead to a heterogeneous cell distribution onto the scaffold. Research in this area either focuses on the development of cell‐free scaffolds seeded with the target cells to form in vitro cardiac tissue or patch,^[^ ^26^, ^27^, ^28^, ^29^ ^]^ or cell‐free scaffolds implanted onto the infarcted myocardium with subsequent recruitment of host cells for cardiac tissue remodeling and regeneration.^[^ ^30^ ^]^ For example, CorMatrix Cor PATCH, an acellular ECM biomaterial derived from porcine small intestinal submucosa, received US FDA approval to be marketed as a patch biomaterial for cardiac reconstruction in 2007 and for vascular repair in 2014.^[^ ^31^ ^]^ Cell‐free scaffolds offer fewer fabrication challenges due to the absence of the cells, allow for removal of toxic chemicals after fabrication, and use of high viscosity materials that can result in more mechanically robust constructs. On the contrary, achieving precise control over the distribution and density of seeded cells onto the scaffolds poses a challenge, with cells frequently adhering only to the surface of the constructs.

### 3.2 Cell‐Laden Constructs

Cell‐laden 3D‐printed scaffolds are fabricated by encapsulating target cells in a hydrogel through deposition of continuous strands in an organized layer‐by‐layer structure.^[^ ^32^, ^33^ ^]^ The main challenge in bioprinting is to ensure the composition of the bioink is 3D‐printable, cytocompatible, and possesses a desirable mechanical strength.^[^ ^33^ ^]^ Hydrogels with high viscosity are more favorable for printability but might not provide a cell‐friendly environment that mimics the physiological properties of ECM. On the other hand, hydrogels with low viscosity are more desirable for cell functions but might not be as mechanically robust as native tissues.^[^ ^33^ ^]^ Cell‐laden constructs can be transplanted either immediately after 3D‐printing^[^ ^17^ ^]^ or be cultured in vitro to allow for cell maturation or differentiation (e.g., stem cells).^[^ ^9^, ^17^, ^34^, ^35^ ^]^

Cell‐laden constructs enable precise control over cell density and distribution throughout the construct, extending beyond the scaffold surface, in contrast to the cell‐free scaffolds. However, the incorporation of live cells into the scaffold fabrication process is challenging. To create a uniform scaffold and prevent cell damage, live cells must be evenly and gently distributed throughout the biomaterial. As well, to optimize results, the bioink must be infused with sufficient nutrients to maintain cell viability during the fabrication process. Often this is achieved by simply mixing the cells with their complete culture media before their addition to the bioink.

### 3.3 Pre‐vascularized Constructs

Transplanted or implanted constructs require a functioning circulatory system to supply nutrients and oxygen to the cells in the construct and to remove cellular waste products.^[^ ^36^ ^]^ The main challenge in this process is the slow growth of vessels. To mitigate this issue, growth factors such as vascular endothelial growth factor or hollow channels created within the scaffolds help encourage ingrowth of host vasculature into the structure.^[^ ^37^ ^]^ Another strategy is to employ in vivo pre‐vascularization with or without the combination of growth factors and microchannels to accelerate the vascularization in tissue scaffolds. 3D‐printed scaffolds, either after maturation in vitro or immediately after printing, are implanted or transplanted in vivo at a site separate from the damaged tissue to allow pre‐vascularization of the scaffold (the body acts as an in vivo bioreactor), with subsequent transplantation into the defect. This technique was effectively employed for tracheal regeneration in rabbits.^[^ ^36^ ^]^ Animals with a more extended period of pre‐vascularization showed more prolonged survival than animals with a shorter or no pre‐vascularization period.^[^ ^38^ ^]^ As well, subcutaneous implantation was used for the purpose of pre‐vascularization in the context of CTE but did not investigate the further transplantation of the pre‐vascularized constructs in animal models of myocardial infarction.^[^ ^11^, ^39^ ^]^ While this approach has potential to provide satisfactory neovascularization, its implementation introduces an additional in vivo step that could raise ethical concerns as well as an increased risk of immunogenicity requiring prolonged use of immunosuppressants. Consequently, studies are attempting to substitute the implantation step necessary for pre‐vascularization with the development of microvessels within the 3D‐printed constructs in vitro before in vivo transplantation.^[^ ^40^ ^]^ Developing pre‐vascularized constructs in vivo allows for the growth and prolonged survival of larger structures, eliminating the necessity for using animals in prevascularization processes.

## 4 In Vitro Characterization Techniques

Tissue‐engineered constructs should possess several key attributes similar to cardiac tissue. These include anatomical and morphological structure comparable to the native one, identical biological and chemical cues, ability to self‐repair and revascularize and to proliferate without the risk of rejection by the body.^[^ ^41^ ^]^ To ensure that the engineered tissue constructs are appropriate for in vivo and clinical studies, an initial round of characterizations should be designed and conducted in vitro. As compared to in vivo ones, in vitro characterizations are cost‐effective, faster, and more appropriate for parameter‐sensitivity examination and analysis in addition to less ethical concerns.^[^ ^42^ ^]^ In vitro characterizations allow for studying various components and properties, such as those associated with biomaterials, fabricated scaffolds, cells within biomaterials, and/or scaffolds.

Prior to fabricating scaffolds using AM techniques, including 3D‐printing, understanding of the material rheology of the biomaterial solution for fabrication is necessary to comprehend its flow behavior. In the case of 3D‐bioprinting, an understanding of the flow behavior of cell‐laden materials is of importance. The flow behavior must be appropriate for printing or bioprinting; too viscous or too elastic solutions might not be extruded in a manner that allows formation of a 3D structure resembling the intended design. Once fabricated, the scaffolds or constructs are examined and characterized in vitro in terms of their physical and biological behavior. For this, the characterizations commonly used include the assessments of physical characteristics (such as swelling and degradation behavior), mechanical properties (such as Young's modulus), structures and morphology (such as pore size and porosity), cell biology and functions (such as cell viability and proliferation), immunostaining and histology, and gene expression analysis. Additionally, properties such as calcium transients, contractility, and excitability hold significance in the context of CTE applications.

The prioritization of these tests depends on the specific application of the cardiac scaffolds. Contractility, calcium transients, and excitability of cardiac scaffolds intended to replace a segment of cardiac tissue need to be examined. This ensures that the transplanted cardiac tissue exhibits rhythmic beating. Also, swelling and degradation of the scaffolds should be synchronized with tissue formation. Additional factors such as mechanical properties and morphology must also harmonize with the biological function of the engineered tissues. Following the conduct of a series of in vitro characterization methods and thorough analysis of the outcomes, a determination can be made regarding the potential of the scaffolds for further in vivo studies.

### 4.1 Rheological Properties

Identification of the rheological properties of a biomaterial is a crucial preliminary step before the development of scaffolds with AM techniques, especially 3D‐printing and electrospinning. Rheological measurements offer invaluable information on a material's behavior throughout the 3D‐printing process, including its ability to be extruded, maintain its shape, and response to stress and strain.^[^ ^43^ ^]^ Therefore, investigation of the rheological characteristics of the printing material, including its shear‐thinning behavior, viscoelasticity, gelling mechanism, and thixotropy, can lead to the development of 3D structures with desired quality and accuracy.^[^ ^43^ ^]^

Fluid behavior is categorized as either Newtonian or non‐Newtonian.^[^ ^43^ ^]^ Newton's law (Equation (1)), where *τ* is the shear stress, *μ* is the viscosity, and $\dot{\gamma }$ represents the shear rate, defines a Newtonian fluid where viscosity remains unaffected by *τ* or $\dot{\gamma }$. Conversely, non‐Newtonian fluids exhibit variable viscosity under specific temperature and pressure, depending on flow conditions including shear rate or flow geometry.^[^ ^43^ ^]^

(1) $$\tau =\mu \dot{\gamma }$$

Non‐Newtonian fluids are categorized into three groups including time‐independent (shear thinning, shear thickening, or viscoplastic), time‐dependent fluids (thixotropic or rheopectic), and viscoelastic.^[^ ^43^ ^]^ While many studies only focus on measuring the viscosity of their ink, viscoelasticity is a key feature for 3D‐printable materials.^[^ ^44^, ^45^ ^]^ A viscoelastic material exhibits an intermediate behavior between an elastic solid and a purely viscous fluid.^[^ ^43^ ^]^ Unlike measuring viscosity, the assessment of viscoelasticity requires oscillatory tests. These tests focus on two primary functions: storage or elastic modulus (*G*′), which reflects the amount of energy that elastically accumulated in the material during its deformation, and the loss or viscous modulus (*G*″), which is a measure of the dissipated energy within the material.^[^ ^43^, ^45^ ^]^ An ideal ink exhibits a balance between elastic and viscous modulus, ensuring high shape fidelity and printing accuracy.^[^ ^45^ ^]^ The rheological behavior of hydrogels can change upon introduction of cells to the bioink depending on the size and density of the cells. This might influence the viscoelastic properties and crosslinking efficiency of the bioink.^[^ ^46^ ^]^

Understanding the rheological behavior of biomaterials/bioinks is imperative before embarking on the design and fabrication of the tissue constructs using AM techniques, including electrospinning and 3D‐printing. This understanding aids in determining the appropriate combination and concentration of biomaterials to achieve high precision and fidelity in printed structures while also ensuring high cell viability during and after 3D‐bioprinting. While there are no standardized rheological characterization methods established for bioinks, numerous studies have examined *G*′, *G*″ for viscoelastic bioinks and the viscosity of purely viscous bioinks. To date, no universally defined and optimized ranges for these moduli and viscosity have been established yet, though some studies recommend the range of 300–30 000 cps for 3D‐printing.^[^ ^47^ ^]^ It is noted that this recommended range is broad and may necessitate adjustments in 3D‐bioprinting conditions based on the specific characteristics of the hydrogel or the type and density of cells within the bioink. Investigating both *G*′ and *G*″ for a bioink can help optimize its rheology using crosslinkers or temperature adjustments (for temperature‐sensitive materials). The goal is to achieve more viscous‐like behavior (*G*″>*G*′) during extrusion and immediately after extrusion, transitioning to more solid‐like behavior (*G*″>*G*′) to stabilize the structure and maintain the living cells within the construct. This will ensure the bioink has an appropriate storage modulus to support cell proliferation within the bioprinted gel.^[^ ^48^ ^]^

In summary, numerous studies in this field have investigated the flow behavior of biomaterial solutions or bioinks (including cells) to understand their responses to various shear stress and shear rate conditions. These studies aim to determine the viscosity or viscoelastic properties of the materials at different frequencies. While this information on the flow behavior is helpful, some studies do not use such information, but trial and error methods to optimize the printing process. In a recent study,^[^ ^49^ ^]^ the rheology of bioink was investigated and used to establish the connections between rheological measurements and the 3D‐printing parameters for use.

### 4.2 Physical Properties

#### 4.2.1 Swelling and Degradation Ratios

Understanding and tuning the swelling and degradation behavior of engineered tissues are important because these properties are among the key elements that affect cell growth and proliferation.

High degrees of swelling might facilitate cell attachment due to enlargement of pores, leading to the distribution of nutrients into the biomaterial from the surrounding media, while a low degree of swelling could result in a decrease in cell attachment and proliferation.^[^ ^50^ ^]^ In a study, a mixture of CMs, CFs, and ECs were encapsulated in alginate/gelatin hydrogel and 3D construct were 3D‐bioprinted.^[^ ^9^ ^]^ Upon in vitro culturing of the constructs, a cardiac tissue model was developed that exhibited sustained longevity and functionality up to 21 days.^[^ ^9^ ^]^ Notably, direct intercellular communication was observed among the cells around days 10–11, peaking on days 20–21. Therefore, the swelling behavior of the scaffolds was assessed over 21 days.^[^ ^9^ ^]^ Their results showed that although the scaffolds sustained 3 weeks of in vitro culture and maintained their macrostructural stability, a significant reduction in their swelling ratio was observed on day 16, indicating the initiation of bulk degradation within the scaffolds.^[^ ^9^ ^]^

As scaffolds act as supporting matrices for cells until a mature and functional tissue forms, scaffolds must undergo gradual degradation over time until they are eliminated from the engineered tissue.^[^ ^51^ ^]^ The degradation rate of a scaffold should be appropriately synchronized for cell growth and proliferation.^[^ ^51^ ^]^ For a too‐early degradation, cells would not have adequate support and protection, which leads to cell death and ultimately the failure of the engineered tissue. For a delayed degradation, suffocation of the proliferating and growing cells may occur, resulting in cell death and even eliciting an immunogenic reaction.^[^ ^51^ ^]^ The degradation test would be conducted similar to the swelling test and would likely be performed on the same samples to enable a direct comparison between the two characterization tests.^[^ ^6^ ^]^

Depending on the application, a high or low swelling and/or degradation ratio might be desired. The controversy arises when hydrogels are considered superior options for use as the main matrix due to their water swelling capacities, allowing them to absorb and maintain nutrients, thereby enhancing cell survival and functionality.^[^ ^52^ ^]^ However, a lower swelling ratio is suggested to be beneficial as it provides more mechanical support for damaged tissue and better integration with the heart wall.^[^ ^53^ ^]^ Although studying the swelling and degradation ratio is commonly used to compare different types of samples, recent studies^[^ ^9^ ^]^ now employ these parameters to link it with structural stability during the experimental period before early signs of tissue formation are observed, which may differ depending on the design of each study.

It is common to obtain the swelling or degradation ratio of the scaffolds while they are incubated in complete culture media at 37 °C.^[^ ^12^, ^53^ ^]^ This typically involves studying the cell‐free scaffolds before incorporating living cells into the constructs. However, this approach only provides information on the swelling and degradation of the materials themselves and does not consider the effects of cellular functioning and metabolic activity. Hence, it is recommended to conduct these tests under sterile condition on constructs that are seeded or encapsulated with the target cells, as this better replicates the physiological conditions.

In conclusion, the appropriate range of swelling and degradation percentages for cardiac tissue is still unknown. These percentages are likely to vary based on factors such as the material used and the type and density of target cells, which affect tissue formation rates. Therefore, it is recommended that the swelling and degradation ratio be monitored until tissue formation occurs and the matrix starts to dissolve completely.

### 4.3 Mechanical Properties

The heart is a mechanically active organ which pumps blood through the circulatory system. The pumping efficiency mainly depends on two properties: the contractility behavior and the passive mechanical properties of the tissue.^[^ ^54^ ^]^ During the past decades, extensive research has focused on understanding and mimicking the mechanics of cardiac contractions. However, there is a growing need to investigate and replicate the passive mechanical characteristics of the heart tissue.^[^ ^54^ ^]^

To the best of our knowledge, the majority of studies on engineered cardiac tissues primarily concentrate on assessing Young's modulus or tensile strength of the scaffolds. The Young's modulus of the native human myocardium is from 10 to 20 kPa at the beginning of diastole and 200 to 500 kPa at the end of diastole. Additionally, the tensile strength of native human myocardium is reported to be 3–15 kPa at the end of diastole.^[^ ^55^, ^56^ ^]^ Although most analyses focus only on static mechanical properties, there are studies reported to studying the dynamic mechanical properties of the engineered tissue or even developing dynamic materials.^[^ ^55^ ^]^ For instance, a study employed dynamic mechanical analysis on scaffolds, starting with a preload force and subsequently applying a force rate ranging from 0.1 N min^−1^ to 1.0 N, followed by a force ramp rate of 0.5 N min^−1^ to 5 N, and finally a force ramp of 1.0 N min^−1^ to 18 N.^[^ ^56^ ^]^ The resulting Young's moduli were determined to be within the range of 40–60 kPa, calculated in the range of 0–10% strain.^[^ ^56^ ^]^ Another study implemented a method involving the fabrication of a substrate constructed from soft poly(dimethylsiloxane) with the capacity for instantaneous stiffening (increasing from 9.3 kPa without magnetization to 54.3 kPa) upon exposure to a magnetic gradient in the presence of cells.^[^ ^55^ ^]^ These scaffolds exhibited variable Young's moduli, with a range close to the beginning of diastole but far from the end of diastole.^[^ ^56^ ^]^

While elucidating the mechanical properties of the scaffold material is of great importance, the emphasis should be directed toward the mechanical behavior of cell‐laden constructs or particularly tissue scaffolds that are grown in vitro. Many analyses focus solely on the mechanical properties of materials before cell incorporation; however, it is evident that cells have a significant impact on Young's modulus. For instance, in cellular scaffolds composed of alginate‐gelatin, the Young's modulus (13476.8 ± 675.6 Pa) was notably higher compared to acellular scaffolds (6405.5 ± 161.8 Pa). This difference highlights the effect of cell presence on the mechanical properties of the scaffolds.^[^ ^9^ ^]^

It is noteworthy that the mechanical properties of both cell‐free and cell‐laden tissue constructs change over time, influenced by factors such as swelling and degradation ratios. For instance, the Young's modulus of 3D‐printed hyaluronan‐based in phosphate‐buffered saline over a 63 day period showed a linear decrease in tensile strength, with the Young's modulus decreasing from 20.5 ± 2.8 to 1.6 ± 2.7 MPa. Additionally, the study indicated that the Young's modulus of precultured tissue scaffolds (cultured in vitro for 3 weeks prior to implantation) was higher upon subcutaneous implantation in mouse models compared to nonprecultured scaffolds.^[^ ^57^ ^]^ However, even for precultured scaffolds, the measured Young's modulus was lower than expected value observed in the in vitro study on the 63 day timepoint. This highlights the significant impact of physiological dynamics compared to the in vitro environment.^[^ ^57^ ^]^

### 4.4 Structural Analyses

The ability of the cells to proliferate and migrate within an engineered tissue scaffold heavily depends on the scaffold's morphology, in addition to its composition and surface chemistry. Scaffolds with proper internal structures create an environment in which nutrients and cellular metabolites can be exchanged freely.^[^ ^58^ ^]^ Therefore, tissue constructs need to be engineered to have sufficient porosity with interconnective pores of adequate size to accommodate cells and their needs.^[^ ^58^ ^]^ Scanning electron microscopy (SEM) and transmission electron microscopy (TEM) techniques are commonly employed tools for studying the morphology of engineered tissues.

#### 4.4.1 SEM and TEM

Both SEM and TEM techniques can be utilized to visualize microstructural morphology of cell‐free scaffolds or cell‐laden constructs. The sample preparation process may differ for in vitro grown tissue constructs compared to cell‐free scaffolds. SEM is recommended for imaging the scaffolds’ surface, with a magnification range of 10‐500 000 times.^[^ ^59^, ^60^ ^]^ TEM enables the study of the internal ultrastructure of cells and tissues, with a magnification range of 2000–1 000 000 times.^[^ ^59^, ^60^ ^]^ SEM and TEM offer invaluable insights into both the surface and internal structures of engineered tissues. However, they operate under vacuum conditions, requiring specific tissue processing that can be potentially destructive to tissues. Managing the risk of sample damage caused by electron beams, particularly in TEM, presents a notable challenge. Therefore, a demand exists for novel and nondestructive techniques that enable the imaging of larger samples with increased thickness.

### 4.5 Cell Viability

Ensuring cell viability on or within 3D engineered tissues is a fundamental requirement for their success. Compared to cell death, defining cell viability is more challenging as it depends on the model and purpose of the study.^[^ ^61^ ^]^ In studies focusing on cytotoxic or cytoprotective effects, evaluating cell viability often involves quantifying the number of viable or proliferating cells or assessing their metabolic activity. However, more complex engineered tissues require multiparametric analyses,^[^ ^61^ ^]^ and the viability is determined by the capability of the cells to differentiate, preserve their heterogeneity, ensure normal turnover, and maintain a 3D pattern.^[^ ^61^ ^]^

Tetrazolium salt MTT (3‐[4,5‐dimethylthiazol‐2‐yl]‐2,5 diphenyl tetrazolium bromide) assay is one the most common colorimetric assays that quantifies cell viability based on active metabolism.^[^ ^61^, ^62^, ^63^ ^]^ MTT is considered a robust reagent metabolized by a wide range of cell types. However, the application of the MTT assay is restricted for engineered tissue constructs due to the limited ability of MTT to penetrate deeply into the cell‐laden 3D construct. Additionally, it functions as an endpoint assay and is limited by its cytotoxic nature, as the ultimate quantification relies on cell lysis. Alternatively, tetrazolium reagent XTT (2,3‐bis(2‐methoxy‐4‐nitro‐5‐sulfophenyl)‐2H‐tetrazolium‐5‐carboxanilide) or resazurin‐based reagents, including Alamar Blue or CellTiter‐Blue, are more cell permeable and less toxic to the cells,^[^ ^64^ ^]^ and may have applicability for cardiac tissue scaffolds. However, these reagents have limitations. While resazurin‐based assays like Alamar Blue offer great sensitivity that can be quantified through either colorimetry or fluorimetry, it is important to be cautious about potential issues. Specifically, at high cell densities, there is a possibility of excessive reduction of Alamar Blue, causing it to transition into the colorless and nonfluorescent hydroresorufin^[^ ^62^ ^]^ which can lead to false results.

Another approach to study cell viability involves the use of live/dead staining protocols.^[^ ^62^ ^]^ In these assays, one reagent marks live cells (often emitting green fluorescence) while another reagent identifies dead cells (often producing red fluorescence). The primary methodology for these kits involves two dyes: one is a membrane‐permeable dye that is metabolized within viable cells (e.g., Calcein AM), and the other is a membrane‐impermeable DNA‐binding molecule (e.g., propidium iodide or ethidium homodimer‐1). After visualizing the samples under a fluorescent microscope, the ratio of live to dead cells can be calculated.^[^ ^62^ ^]^

In summary, the choice of cell viability assay should align with the target cells and tissue dimensions. For assessment of large‐sized 3D engineered tissue, an appropriate colorimetric or fluorometric assay must meet two key criteria: 1) The compound used in the assay should be capable of freely diffusing into the engineered tissue, undergoing metabolism, and subsequently exiting the tissue. This enables reliable readings to be taken from the culture media encompassing the tissue construct.^[^ ^64^ ^]^ 2) The assay should show minimal cytotoxicity and could be washed out after the measurement. This characteristic permits multiple and repetitive measurements on the same tissue construct over time.^[^ ^64^ ^]^ However, the majority of cell viability assays, including MTT and live/dead assays commonly used in CTE studies, are endpoint assays, making it challenging to monitor cell viability continuously throughout the experimental period.

### 4.6 Histological Analyses

Histological analyses are a gold standard for tissue examination, including assessment of inflammation and healing. It is also a valuable tool to identify the presence and distribution of degradation products infiltrated into the surrounding tissues.^[^ ^65^ ^]^ Specific structures, cells, and tissues could be identified by appropriate markers.^[^ ^65^ ^]^ While histological analyses are commonly performed on harvested samples from animals or humans, these analyses can also be conducted on engineered tissues that have been cultivated in vitro.

#### 4.6.1 Histology

**Figure 2:**
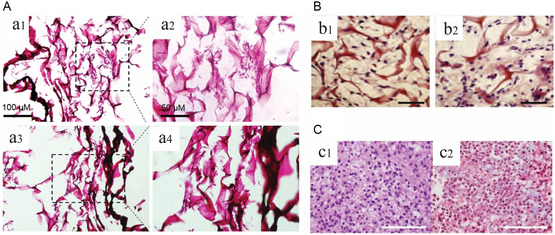
Histological images of engineered cardiac tissues captured by light microscopy. A) H&E staining to confirm the penetration of human cardiac stem cells inside the scaffolds composed of (a1–a2) ECM with polysaccharides at a ratio of 75:25 (E75/P25) and (a3–a4) ECM only. Reproduced with permission.^[^ ^70^ ^]^ Copyright 2020, Elsevier. B) H&E staining to confirm the biocompatibility of CMs loaded *Antheraea mylitta* silk fibroin scaffolds on (b1) day 8 and (b2) day 20 (scale bar: 50 μm*)*. Reproduced with permission.^[^ ^69^ ^]^ Copyright 2012, Elsevier. C) H&E staining (c1) and Masson trichrome (c2) of bioprinted cardiac patches to confirm presence of the cells and fibrous tissue in the matrix (scale bar: 100 μm). Reproduced with permission.^[^ ^71^ ^]^ Copyright 2017, Nature. (license: http://creativecommons.org/licenses/by/4.0/.).

Histochemistry combines biochemical and histological techniques that use tissue sections as the medium in which biochemical reactions are initiated and conducted by adding specific substrates. The histochemical reactions localize cell structures, expressing a particular property.^[^ ^66^, ^67^ ^]^ Histological analyses typically are conducted on harvested scaffolds, both with or without the surrounding tissues, or on tissues after scaffold degradation within in vivo models. In the context of CTE, Masson trichrome^[^ ^17^, ^28^ ^]^ and hematoxylin and eosin (H&E) stains are commonly used.^[^ ^17^, ^68^ ^]^ **Figure** **2** illustrates representatives of histological staining on engineered cardiac tissues. These stains can help determine whether the cells penetrate the scaffolds, resulting in highly cellular constructs, and assess scaffold biocompatibility.^[^ ^15^, ^69^, ^70^, ^71^ ^]^

While histological analyses are primarily employed for examining tissue biopsies or engineered tissue samples post implantations/transplantation, there is a growing need to extend the use of these analyses to in vitro grown tissues, especially engineered tissues.

#### 4.6.2 Immunostaining

Immunostaining is a tool to identify the progressive development of engineered cardiac tissue. This technique can assess whether the engineered cardiac tissue was formed with electromechanically coupled and uniformly aligned and dense cardiac cells.^[^ ^34^ ^]^ The primary antibody (also target antigen) is localized and visualized via microscopy by employing a colloidal gold, enzymatic, biotin, or fluorescent labeled detection system.^[^ ^72^, ^73^, ^74^ ^]^ Enzyme‐based labels are visible with a light microscope.^[^ ^73^ ^]^ Colloidal gold labels are visualized with an electron microscope.^[^ ^73^ ^]^ A fluorophore can be visualized with a fluorescent microscope, while biotin labels can be visible with light, fluorescence, and electron microscopy combined with avidin–biotin complex techniques.^[^ ^73^ ^]^

Immunohistochemistry is a powerful means for uncovering the tissue distribution of an antigen of interest.^[^ ^72^ ^]^ The choice of fixation method, washing, selecting the proper antibodies, incubation conditions, and the appropriate mounting of cells and tissues onto slides are essential to immunohistology.^[^ ^75^, ^76^ ^]^ Antigen–antibody binding is the core of all immunohistochemical protocols, which may result in good quality immunohistochemical staining upon binding of the primary antibody.^[^ ^77^ ^]^

The choice of the appropriate fixation could be made by trial and experience because no universal ideal fixation methods exist in immunohistochemistry.^[^ ^76^ ^]^ The amino acid sequence (epitope) recognized by the antibody may be hidden, modified, or even lost due to an inadequate fixation.^[^ ^76^ ^]^ Fixing the samples in formalin and embedding them in paraffin is one of the most common ways to prepare them for immunohistochemistry analyses.^[^ ^23^ ^]^ It provides excellent morphology but significantly modifies proteins.^[^ ^75^ ^]^ Flash‐freezing sections is beneficial in terms of maintaining tissue structure and delivering immediate results.^[^ ^75^ ^]^ However, tissue morphology is not as well preserved as paraffin‐embedded sections, and it requires a high degree of technical skill. Another method is embedding tissue in polyester wax, leading to intermediate results between paraffin‐embedded sections and frozen sections regarding morphology and antigenicity.^[^ ^75^ ^]^

**Figure 3:**
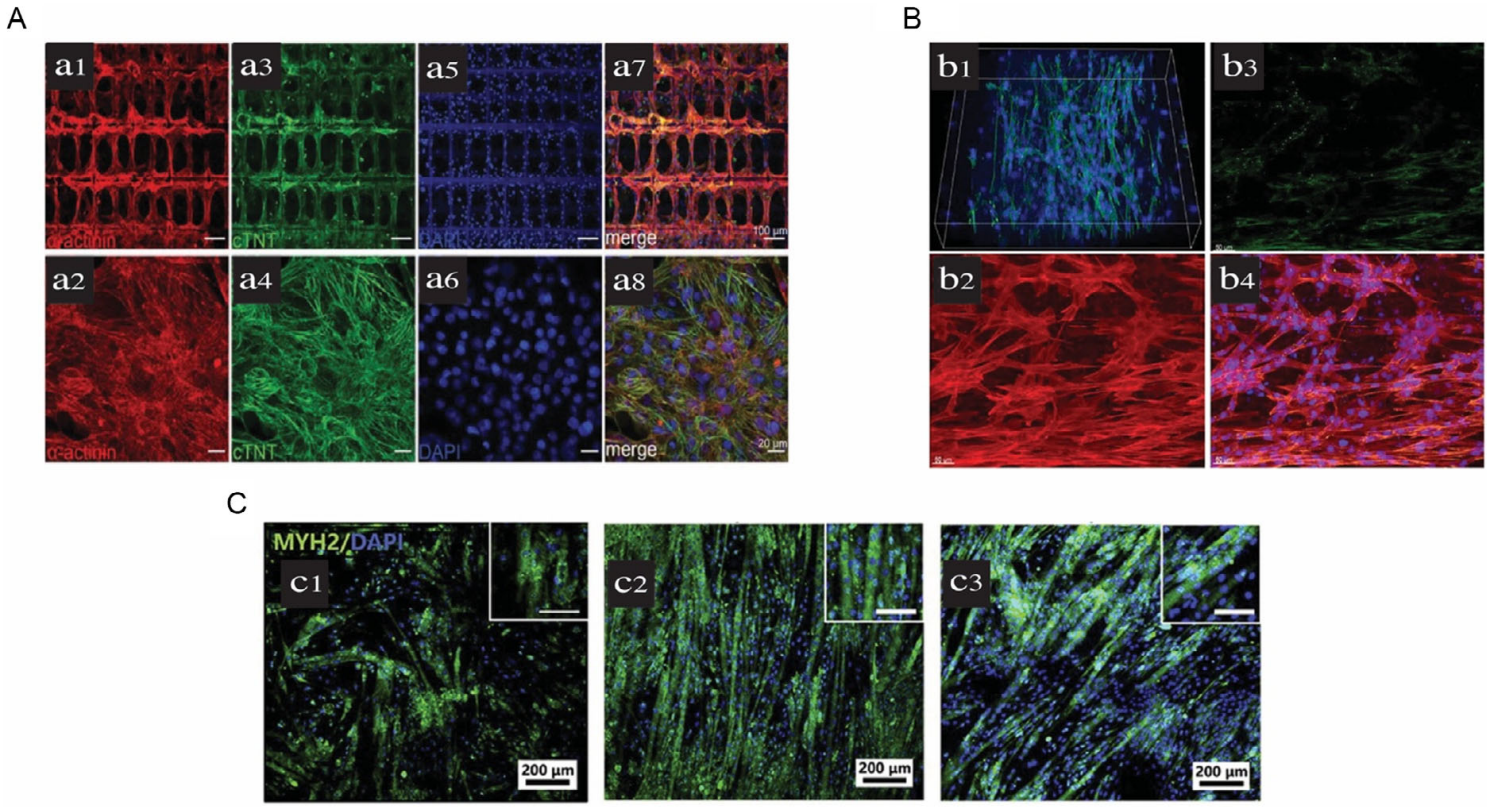
Immunofluorescence staining of different engineered cardiac tissues. A) 3D‐printed constructs seeded with human embryonic cell‐derived cardiomyocytes (hiPSC‐CMs) after 10 days of in vitro culture stained with α‐actinin (a1 and a2) and cardiac troponin T (cTNT, a3 and a4), and DAPI (a5 and a6). (a7) and (a8) Represent the merged images, indicating a promoted striation pattern of the engineered tissues with regularly arranged myofibrils along one long axis. Reproduced with permission.^[^ ^27^ ^]^ Copyright 2023, John Wiley and Sons. (licence: https://creativecommons.org/licenses/). B) Scaffold‐free 3D‐printed model for cardiac cells embedded in fibrin‐based biomaterial after 7 days of in vitro culture stained with Cx43 antibody and Hoechst (b1), single plane images corresponding to (b1): F‐actin alone (b2), Cx43 alone (b3), and merge of all channel (b4). The staining shows the cardiac cellular morphology that mimic native cardiac cell culture as well as overexpression of CX43 due to enhanced functional organization of cardiac cells within the engineered cardiac tissue. Reproduced with permission.^[^ ^48^ ^]^ Copyright 2022, Elsevier. C) Electrospun scaffolds 6 days after in vitro incubation in differentiation media stained with MYH2 (green) and DAPI (blue) composed of (c1) PLA, (c2) PLA/PANI1.5, and (c3) PLA/PANI3. The images demonstrate the impact of conductivity on the maturity of myotubes, revealing that more mature myotubes were formed on PLA/PANI1.5 and PLA/PANI3 compared to PLA nanofibrous sheets. Reproduced with permission.^[^ ^78^ ^]^ Copyright 2017, Elsevier.

The choice of antibodies depends on the focus of the study, with several proteins being crucial for investigating the development of functional cardiac tissue. Sarcomeric α‐actinin serves as a vital marker for studying CMs maturation and contractility.^[^ ^78^ ^]^ Additionally, connexin 43 (CX43), a gap junction protein, plays a significant role in cell–cell coupling, ensuring synchronous beating of CMs.^[^ ^78^ ^]^ Targeting filamentous actin (F‐actin), which forms the thin filaments in the myofibrils of muscle cells, is essential due to its involvement in various critical processes such as cell division, migration, morphogenesis, intracellular cargo transport, and muscle contraction.^[^ ^79^ ^]^ Phalloidin is commonly employed as the standard marker for labeling F‐actin in fixed samples and tissue.^[^ ^79^ ^]^ Cardiac troponin T (cTnT) and troponin I (cTnI) represent cardiac regulatory proteins primarily exclusive to the myocardium, with the exception of cTnI, which might exhibit limited expression in skeletal muscle.^[^ ^80^ ^]^ These proteins play a crucial role in regulating the calcium‐mediated interaction between actin and myosin.^[^ ^80^ ^]^ To further investigate the myogenic differentiation of CMs, myosin heavy chain 2 (MYH2) can be labeled, and parameters such as the length, diameter, quantity, maturation, and fusion indices of myotubes can be measured.^[^ ^78^ ^]^ **Figure** **3** illustrates examples of engineered cardiac tissues labeled with different antibodies to detect specific proteins, including α‐actinin and cTnT (Figure 3A), Cx43 and F‐actin (Figure 3B), and MYH2 (Figure 3C).

**Table** **3** also summarizes a few common reagents used for immunostaining assays conducted on engineered cardiac tissues. Many studies use either DAPI ((4′,6‐diamidino‐2‐phenylindole) or Hoechst for staining nucleus.

**Table 3:** Fluorescent labels and antibodies employed in immunofluorescent assays on engineered cardiac tissues.

Primary antibodies/fluorescent labels	Purpose	References
Phalloidin F‐actin	To detect CX43 (a gap junction protein that facilitates electrical coupling between CMs) and phalloidin F‐actin	[48]
CX43		
MYoH	To detect CX43 and myosin heavy chain (MyoH) in HL‐1 cells and detecting PECAM‐1 (also known as CD‐31) in ECs	[9]
CX43		
CD‐31		
Tetramethylrhodamine B isothiocyanate (TRITC)	To stain the actin cytoskeleton in CMs (H9c2 cells)	[112]
α‐Sarcomeric actinin	To detect α‐sarcomeric actinin and troponin‐t (associated with cell contraction)	[87]
Cardiac troponin T		
ActinGreen 488 ReadyProbes Reagent	To stain F‐actin and the cell nucleus	[113]
NucBlue Fixed Cell ReadyProbes Reagent		
α‐Actinin	To study two specific markers of mature and contractile CMs: α‐actinin (a sarcomeric protein) and CX43 (a gap junction protein involved in electrical coupling among CMs)	[114, 115]
CX43		
Cardiac myosin heavy chain IgG	To label β‐MHC and troponin T (the mature markers that reflect differentiation of CMs) and detect vinculin (a focal adhesion protein used for visualizing the spatial distribution of focal adhesions within the cells) as well as detecting F‐actin filaments anisotropy and orientation	[29]
cTnT		
Vinculin IgG		
Rhodamine phalloidin dye		
NKX2‐5	To investigate the expression of the cardiac specific proteins	[116]
cTnT		
Actin		
CX43		
Sarcomeric α‐actinin		
Myosin heavy chain 2 (MYH2)	To detect MYH2 to check for myotube formation in H9c2 cells, as well as labeling CX43 (a protein responsible for cell communication and synchronous beating of CMs) and α‐actinin (a protein responsible for CMs maturation and their strong contractility)	[78]
α‐Actinin		
CX43		
cTnT	To study the expression of CMs, smooth muscle and ECs.	[28]
α‐Smooth muscle actin (α‐SMA)		
CD‐31		
Sarcomeric α‐actinin	To label CM‐specific markers (sarcomeric α‐actinin, CX43, and cardiac troponin I) and also to check the pluripotency of iPSCs (NKX 2.5)	[16]
CX43		
cTnI		
NKX 2.5		
cTnI	To assess how the cells are integrated and organized within the remodeled matrix.	[36]
Collagen type I		
Ventricular myosin light chain 2 (MLC2v)		

Although valuable information could be obtained through labeling various proteins, current immunofluorescence assays do not enable dynamic evaluation of these proteins. Some information may be lost or obscured during the fixation, embedding, sectioning, and staining of cells. Additionally, limitations arise in targeting only a few antibodies on each sample due to potential interactions among the employed antibodies. Therefore, there is a need for more advanced procedures that enable real‐time expression of multiple proteins within engineered cardiac tissue.

### 4.7 Gene Expression

Each human tissue carries out its own gene expression patterns that is controlled by specific regulatory programs.^[^ ^81^ ^]^ Therefore, investigating the expression of specific genes after culturing engineered tissue in vitro could offer valuable insights toward the formation and functionality of newly developed tissue.

Several genes, encoding specific proteins for a functional engineered cardiac tissue, are commonly examined in various studies on developing engineered cardiac tissues. These include: 1) *TNNT2*: Encodes cTnT and regulates muscle contraction through changes in intracellular calcium ion concentration.^[^ ^82^, ^83^ ^]^ 2) *GJA1*: Encodes the CX43 protein and it is responsible for regulating gap junctions and intercellular coupling in myocardial tissue.^[^ ^82^, ^83^ ^]^ 3) *MYH6*: Encodes α‐myosin heavy chain (α‐MyHC) and it is a vital component of thick filaments.^[^ ^82^, ^83^ ^]^ 4) *MYH7*: Encodes β‐myosin heavy chain (β‐MyHC) and it is another crucial component of thick filaments.^[^ ^82^, ^83^ ^]^ 5) *ATP2A2*: Encodes one of the SERCA Ca (2+)‐ATPases and it is a vital component involved in excitation/contraction coupling of CMs.^[^ ^82^ ^]^

While immunohistological analyses can label proteins of interest to study the functionality of engineered cardiac tissue, examining the expression of genes associated with cardiac functions allows for a quantitative analysis of the expression levels of representative markers. For instance, the effect of patterned and/or conductive substrates on CMs development was quantitatively analyzed using qRT‐PCR.^[^ ^84^ ^]^ Cells showed an increased expression of *hMYH7* when cultured on patterned and/or conductive substrates compared to nonpatterned and nonconductive counterparts, while *TNNT2* expression only increased when the cells were cultured on conductive substrates and remained unaffected by substrate topography.^[^ ^84^ ^]^ Although numerous studies within CTE investigate the expression of genes associated with cardiac functions, there is a critical need for a more focused exploration of understanding and manipulating genes in vitro to enhance functionality in engineered cardiac tissue. Further research is warranted to investigate the link between gene expression and maturation of engineered cardiac tissue.

### 4.8 Functional Analyses: Calcium Imaging, Contractility, and Excitability

A key parameter of engineered cardiac tissue is the ability to generate comparable contractile forces with human heart tissue.^[^ ^85^ ^]^ However, interpretation of force data is complicated as many factors affect the generation of contractile force such as the proteins responsible for handling calcium.^[^ ^85^ ^]^ Given the importance of calcium, an approach was developed whereby readouts of cytoplasmic calcium concentrations (calcium transient, CaT) are combined with force analysis to obtain more meaningful interpretation of contractility.^[^ ^85^ ^]^

**Figure 4:**
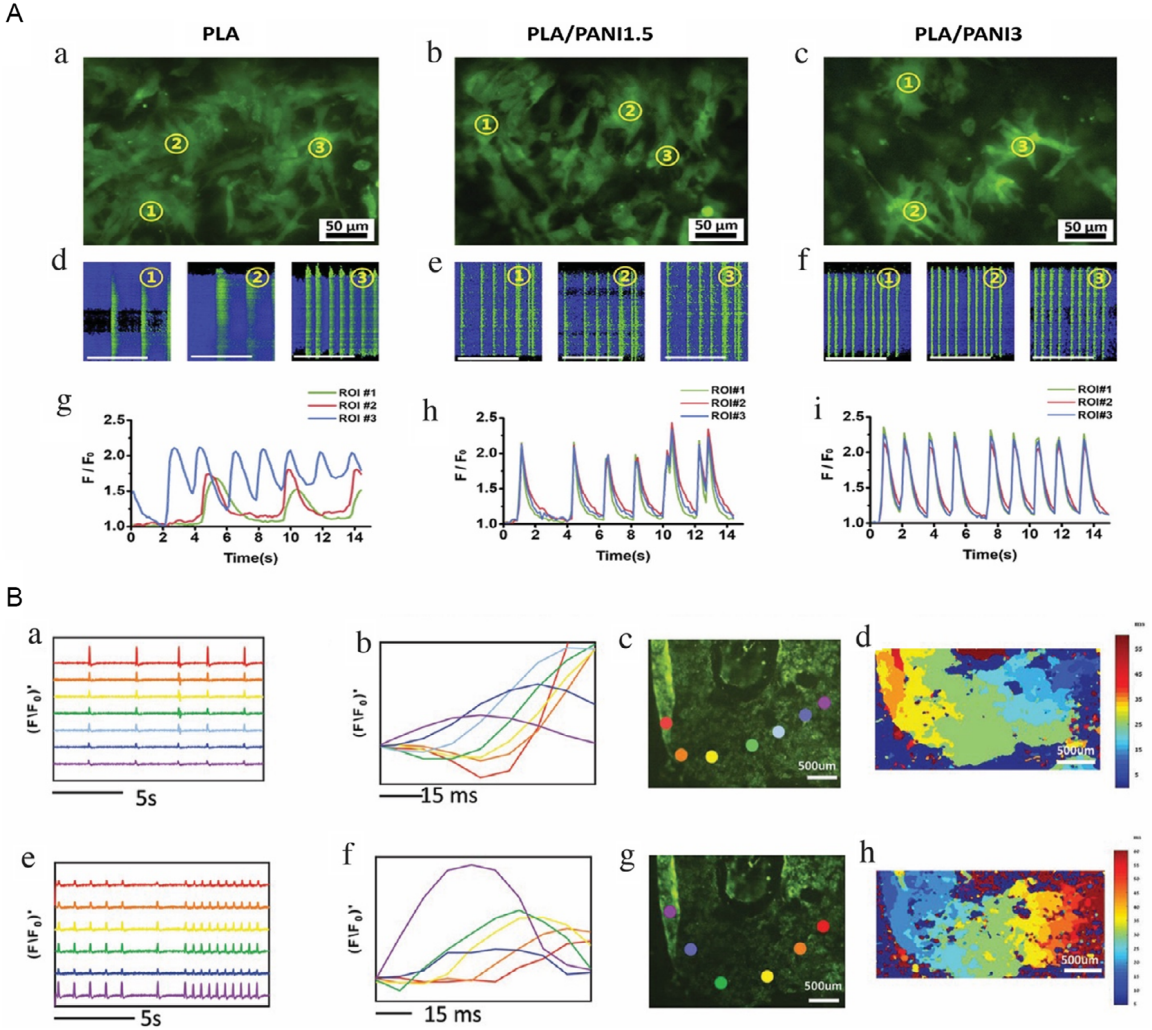
Calcium transients imaging on A) PLA, PLA/PANI1.5, and PLA/PANI3 nanofibrous sheets. ROIs were randomly assigned in a) PLA, b) PLA/PANI1.5, and c) PLA/PANI3 groups. (d–f) Spontaneous calcium transients are shown through line scanning across each ROI (scale bar:10 s). (g–i) F/F0 representing the normalized fluorescence intensity of each ROI. Reproduced with permission.^[^ ^78^ ^]^ Copyright 2017, Elsevier. B) Nanoengineered peptide‐based conductive biomaterial. (a–d) Calcium transient imaging analysis for seven assigned ROI. (e–h)Calcium transient imaging analysis for each ROI in the presence of an electrical stimulation. (a,d) F/F0 for each ROI; (b,f) Calcium flux delay between different ROI; (c,g) The seven separate sites representing each ROI; (d,h) Heat map of propagation of Ca2+ signal. Reproduced with permission.^[^ ^87^ ^]^ Copyright 2021, Wiley & Sons.

Calcium (Ca^2^+) signaling is involved in several functions of CMs, including controlling each cycle of contraction and relaxation. This process involves transient increases and decreases in cytosolic calcium (Ca^2^+), which are facilitated by the sarcoplasmic reticulum calcium ATPase (SERCA2) and other proteins.^[^ ^86^ ^]^ Therefore, a way to investigate the functionality of engineered cardiac tissues is through analysis of the Ca^2^+ transient imaging.^[^ ^87^ ^]^ The engineered tissue is incubated with a calcium‐sensitive dye, such as Fluo‐4AM, a few days after its development. Then, a few regions are selected, and using a fluorescent microscope, images and/or videos are acquired and analyzed with software such as ImageJ and MATLAB.^[^ ^78^, ^87^ ^]^ Once the images and/or videos are obtained, several independent regions of interest (ROIs) are randomly chosen for analysis. The findings are typically presented as F/F_0_, where F denotes the fluorescence intensity at intermediate calcium concentrations, and F_0_ represents the baseline fluorescence intensity. **Figure** **4** represents two instances of Ca^2^+ transient imaging conducted on engineered cardiac tissues. Spontaneous calcium transients, showing calcium sparks during beating, were captured.^[^ ^78^ ^]^ The results showed asynchronous calcium transients in the polylactic acid (PLA) group and synchronized calcium transients in three independent ROIs for sheets containing polylactic acid/polyaniline (PLA/PANI), including both PANI1.5 and PANI3. This synchronization resulted in enhanced coupling among CMs during spontaneous beating^[^ ^78^ ^]^ (Figure 4A). These results are consistent with another study that developed a peptide‐based hydrogel containing PANI. In that study, spontaneous and synchronous contraction of CMs across the hydrogel surface was recorded, demonstrating the patch's ability to support functional cell growth and the formation of electrically coupled CMs^[^ ^87^ ^]^ (Figure 4B).

Although current Ca^2^+ transient imaging techniques provide insightful information about the functionality and contraction of CMs, they are limited to studying Ca^2^+ transients on the surface of engineered patches and seeded cells. There is a need to enhance these techniques to record Ca^2^+ transients within 3D structures, especially for 3D‐bioprinted constructs. Additionally, the lack of standardized protocols for calcium transient imaging makes it difficult to compare results across different studies.

While Ca^2^+ transient imaging provides indirect information about the contractility of heart muscle, direct measurement of contractility should also be studied. Once the cells within the construct start to contract, their motion can be recorded. The beating rates, including contraction, relaxation, and beats per minute, can then be computed using an open‐source video analysis algorithm.^[^ ^35^ ^]^ There are novel techniques that allow for synchronous measurements of Ca^2+^ and force for characterization of excitation–contraction coupling in human myocardium.^[^ ^88^ ^]^

Rhythmic excitability is a crucial property for engineered cardiac tissues. This excitability enables each cardiac cell to contract and relax, generating an action potential at their membranes in response to cardiac depolarization and repolarization.^[^ ^88^ ^]^ Depolarization of CMs leads to Ca^2^+ release from the sarcoplasmic reticulum, causing myofibril contraction and subsequently cardiac muscle contraction.^[^ ^89^ ^]^ Therefore, electrical stimulation appears essential to train CMs.^[^ ^89^ ^]^ Additionally, the electrical activity of the CMs in engineered cardiac tissue should be thoroughly studied. The multielectrode arrays (MEA) could be employed for measuring the electrical activity of CMs, allowing for the simultaneous monitoring of electrical activity at multiple sites.^[^ ^90^ ^]^ In MEA, electrodes are distributed over a small surface area (on the millimeter scale) where cells in a monolayer or within engineered cardiac tissue can be directly cultured.^[^ ^89^ ^]^ This approach is suitable for evaluating extracellular field potential (EFP), heartbeat frequency, repolarization properties, conduction velocity, and trajectories, facilitating noninvasive and long‐term exploration of the electrophysiological features of both individual CMs and cells within the tissue.^[^ ^89^ ^]^ After the maturation of CMs and once the MEA sensors come into contact with the engineered cardiac tissue, the generation of an action potential by the tissue produces a transient transmembrane potential and ionic current. This activity polarizes the electrodes, altering their potential.^[^ ^91^ ^]^ Consequently, the electric signal is amplified and recorded as an EFP.^[^ ^91^ ^]^ It has been demonstrated that MEA sensor can record a stable signal of engineered cardiac tissue.^[^ ^91^ ^]^ However, this method is limited to measuring small areas detectable by the electrodes and does not offer comprehensive information about the propagated action potentials.^[^ ^89^ ^]^ Therefore, developing more advanced technology to study the functionality of cardiac patches, including Ca^2+^ transients, contractility, and excitability, seems necessary.

In summary, depending on the application and duration of usage of the cardiac patch, decisions should be made regarding the selection and optimization of appropriate materials, cells, and fabrication techniques. Once the tissue is engineered, all necessary characterizations should be performed in vitro before proceeding with in vivo and/or clinical studies. **Table** **4** provides a concise overview of selected studies in CTE, detailing the fabrication techniques, biomaterials, cells, and characterization methods employed in each study.

**Table 4:** Highlight of studies of biomaterials, cells, scaffold fabrication techniques, and in vitro characterization methods for CTE.

Scaffold fabrication technique	Biomaterials	Cells	Cells incorporation technique	In vitro characterizations	Outcomes	References
3D‐printing	Alginate, gelatin	HUVECs, H9c2	Seeding	Printability, cell viability, Young's modulus, swelling and mass loss percentage, 2D and 3D structural analysis (SEM, SR‐PBI‐CT)	High viability of seeded cells on the scaffolds, 3D‐printed with a combination of alginate and gelatin (3:1 ratio) and high Young's modulus	[6]
3D‐printing	Polylactic acid	hiPSC‐derived CMs	Seeding	Printability, simulation, morphological and immunohistochemical analysis, calcium imaging	Formed synchronous functional and ordered engineered cardiac tissue with grown sarcomeres along the fiber	[27]
3D‐bioprining	Alginate, fibrinogen	A16, NVRM	Encapsulation in bioink	Printability, Fourier transform infrared (FTIR), rheology, tube inversion method, pore factor, SEM, elastic modulus and percentage elongation at break, degradation and swelling, live/dead assay, MTS assay, immunofluorescence	High NRVM viability (>80%) and sarcomeric alpha actinin & connexin 43 expression within the 3Dbioprinted construct	[10]
3D‐bioprining	GelXA Laminink‐521 bioink	hiPSCs (from skin)	Encapsulation in bioink	Contraction dynamics, immunostaining, gene expression	Viable CMs with spontaneous contraction for 30 days and appropriate phenotype and function, progressive and enhanced maturation of CMs within the 3D tissue construct compared to 2D culture	[117]
3D‐bioprinting	Alginate, gelatin	AC16,CFs,ECs	Encapsulation in bioink	Swelling and degradation, ATR‐FTIR, rheological analysis, cell biocompatibility and immunostaining analysis	High viability and proliferation of cell mixtures over 21 days following 3D‐bioprinting, confirmed cardiac‐specific cell functionality	[9]
3D‐bioprinting	Gelatin methacrylate, collagen methacrylate, fibronectin, laminin‐111, lithium phenyl‐2,4,6‐trimethylbenzoylphosphinate (photocrosslinker)	hiPSC	Encapsulation in bioink	Bioink viscosity determination, cell viability and proliferation, cell density, rheological analysis, print fidelity, dye perfusion, immunohistochemical assessment, calcium transient measurement, optical mapping macroscale beating video acquisition, contractility, pressure/volume assessment, *in silico* finite element model, inverted geometry with filling, MRI and anatomical fidelity analysis, perfusion test, cell viability	Human chambered muscle pumps with macroscale beating and continuous action potential propagation	[35]
3D‐bioprinting	Decellularized omentum	iPSC‐derived CMs, HUVECs	Encapsulation in bioink	Mathematical modeling, perfusion, calcium imaging, immunostaining	Generated a reinforced engineered cardiac tissue with fully biocompatibility, Fully cellularized, vascularized, and thick engineered cardiac tissue	[118]
Electrospinning	Polycaprolactone, gelatin	hiPSC, HL‐1	Seeding	Cell proliferation, contraction analysis, immunostaining, gene expression, enzyme‐linked immunosorbent assay (ELISA)	Elongated morphology of hiPSC‐CMs, Extended hiPSC‐CMs along the fiber axis, enhanced sarcomere organization, spontaneous synchronous contraction, matured hiPSC‐CMS	[83]
Electrospinning	Furfuryl‐gelatin, polycaprolactone	A16, iPSC‐CMs	Seeding	Structural analysis (SEM, TEM), FTIR, thermogravimetric analysis (TGA), differential scanning calorimetry (DSC), rheological analysis, swelling, adhesion test using confocal imaging, cell biocompatibility and proliferation, live/dead assay	High biocompatibility (>90%) of AC16 cells, high elastic modulus, adhesion and functionality of hiPSC‐CMs	[119]
Electrospinning	Gelatin, poly(lactic‐*co*‐glycolic) acid, polypyrrole	NRVM, iPSC, and human lung fibroblasts (MRC‐5)	Seeding	Morphology and fiber alignment (SEM), water contact angle, shrinkage, swelling, porosity, degradation rate, electrical characterizations, mechanical properties, cell viability and morphology, gene expression analysis	More mature NRVMs following seeding on aligned conductive construct compared to nonaligned construct	[120]

## 5 Conclusions and Future Perspectives

CTE has been drawing considerable attention nowadays for repairing cardiac tissue damage and/or replacing the fibrotic scar tissue, where the scaffold fabrication and in vitro characterization are the first, yet key step, to its success in in vivo. In this article, we briefly review the biomaterials, cells, and fabrication techniques involved in the development of scaffolds for CTE, as well as the in vitro characterization techniques and/or methods essential to ensure the success in the in vivo implantation experiments.

The biomaterials used to develop the 3D structure of myocardium should mimic the heart's ECM. Alginate‐based hydrogels are a popular choice due to their cost‐effectiveness and biocompatibility; however, alginate alone does not support fibronectin adhesion and thus needs to be combined with other materials. Heart tissue‐derived ECM is a more recent and promising option that closely mimics the heart's microenvironment, despite challenges such as the presence of residual DNA that need to be overcome.

Among the three different categories of cells employed in CTE—immortalized cell lines, primary cells, and stem cells—stem cells, especially induced pluripotent stem cells (iPSCs), seem the most promising option due to their suitable properties, such as pluripotency, proliferative capacity, and accessibility.

AM technologies, including 3D‐printing and electrospinning, enable the fabrication of tissue constructs using top‐down or bottom‐up approaches. These methods allow for the creation of complex structures, such as myocardium. Depending on the application of the developed cardiac tissue, the tissue can be designed and manufactured through three different approaches: cell‐free scaffolds, cell‐laden constructs, and pre‐vascularized constructs.

Following the development of the cardiac scaffolds, a series of in vitro examinations becomes essential before initiating in vivo studies. These include evaluation of the physical properties of the cardiac constructs, such as their swelling and degradation behavior over time, examination of their mechanical characteristics, such as Young's modulus, microscopic evaluation of the structure of the constructs with techniques such as SEM and TEM, and the conduct of cell biology assays to gauge cell viability and proliferation within the tissue construct. Immunohistological analyses, functional analyses including calcium imaging, contractility, excitability, and gene expression analyses are also essential to ensure the development of cardiac tissue. Notably, each characterization technique has its limitations, particularly because most are designed for native tissues rather than engineered scaffolds. Therefore, it is recommended to establish a universal and standardized set of characterization techniques tailored specifically for engineered tissues. Additionally, the use of dynamic environments, such as bioreactors that mimic the complex microenvironment of the heart, is necessary for the growth and characterization of these tissues. Furthermore, the development of cardiac tissues should focus on personalized medicine, with cardiac constructs customized to meet each patient's needs and derived from their own biological materials. Finally, tissue engineering approaches should be integrated with artificial intelligence technologies to incorporate complex datasets and better predict the performance of engineered tissues.

## Conflict of Interest

The authors declare no conflict of interest.

## Author Contributions

**Farinaz Ketabat**: was responsible for Conceptualization; Writing—original draft; Writing—review and editing. **Jane Alcorn**: was responsible for Writing—review and editing. **Michael E. Kelly**: was responsible for Conceptualization; Funding acquisition; Supervision; Writing—review and editing. **Ildiko Badea**: was responsible for Conceptualization; Funding acquisition; Supervision; Writing—review and editing. **Xiongbiao Chen**: was responsible for Conceptualization; Funding acquisition; Resources; supervision; and Writing—review and editing.
